# Empagliflozin and Cerebrovascular Events in Patients With Type 2 Diabetes Mellitus at High Cardiovascular Risk

**DOI:** 10.1161/STROKEAHA.116.015756

**Published:** 2017-04-24

**Authors:** Bernard Zinman, Silvio E. Inzucchi, John M. Lachin, Christoph Wanner, David Fitchett, Sven Kohler, Michaela Mattheus, Hans J. Woerle, Uli C. Broedl, Odd Erik Johansen, Gregory W. Albers, Hans Christoph Diener

**Affiliations:** From the Lunenfeld-Tanenbaum Research Institute, Mount Sinai Hospital, Toronto, Canada (B.Z.); Division of Endocrinology (B.Z.) and St Michael’s Hospital, Division of Cardiology (D.F.), University of Toronto, Canada; Section of Endocrinology, Yale University School of Medicine, New Haven, CT (S.E.I.); The Biostatistics Center, The George Washington University, Rockville, MD (J.M.L.); Comprehensive Heart Failure Center and Renal Division, University of Wuerzburg and Hospital, Germany (C.W.); Boehringer Ingelheim Pharma GmbH and Co. KG, Ingelheim, Germany (S.K., M.M., H.J.W., U.C.B.); Boehringer Ingelheim Norway KS, Asker (O.E.J.); Department of Neurology and Neurological Sciences, Stanford Stroke Center, CA (G.W.A.); and Department of Neurology and Stroke Center, University Hospital Essen, Germany (H.C.D.).

**Keywords:** blood pressure, cardiovascular diseases, hematocrit, stroke, type 2 diabetes mellitus

## Abstract

**Background and Purpose—:**

In the EMPA-REG OUTCOME trial (Empagliflozin Cardiovascular Outcome Event Trial in Type 2 Diabetes Mellitus Patients), empagliflozin added to standard of care in patients with type 2 diabetes mellitus and high cardiovascular risk reduced the risk of 3-point major adverse cardiovascular events, driven by a reduction in cardiovascular mortality, with no significant difference between empagliflozin and placebo in risk of myocardial infarction or stroke. In a modified intent-to-treat analysis, the hazard ratio for stroke was 1.18 (95% confidence interval, 0.89–1.56; *P*=0.26). We further investigated cerebrovascular events.

**Methods—:**

Patients were randomized to empagliflozin 10 mg, empagliflozin 25 mg, or placebo; 7020 patients were treated. Median observation time was 3.1 years.

**Results—:**

The numeric difference in stroke between empagliflozin and placebo in the modified intent-to-treat analysis was primarily because of 18 patients in the empagliflozin group with a first event >90 days after last intake of study drug (versus 3 on placebo). In a sensitivity analysis based on events during treatment or ≤90 days after last dose of drug, the hazard ratio for stroke with empagliflozin versus placebo was 1.08 (95% confidence interval, 0.81–1.45; *P*=0.60). There were no differences in risk of recurrent, fatal, or disabling strokes, or transient ischemic attack, with empagliflozin versus placebo. Patients with the largest increases in hematocrit or largest decreases in systolic blood pressure did not have an increased risk of stroke.

**Conclusions—:**

In patients with type 2 diabetes mellitus and high cardiovascular risk, there was no significant difference in the risk of cerebrovascular events with empagliflozin versus placebo.

**Clinical Trial Registration—:**

URL: http://www.clinicaltrials.gov. Unique identifier: NCT01131676.

## Abstract 2

Supplemental Digital Content is available in the text.

Patients with diabetes mellitus are at increased risk of cardiovascular events and cardiovascular mortality.^1^ The risk of stroke in patients with diabetes mellitus is increased 2-fold compared with individuals without diabetes mellitus^1^; the risk of recurrent stroke is also increased.^2^ Trials of intensive glucose-lowering^3^ or of specific glucose-lowering agents,^4–7^ with the exception of pioglitazone^8^ and semaglutide,^9^ have not been shown to significantly reduce the risk of stroke in patients with type 2 diabetes mellitus even after prolonged follow-up.

Empagliflozin is a potent and selective inhibitor of SGLT2 (sodium glucose cotransporter 2) used in the treatment of type 2 diabetes mellitus. In the EMPA-REG OUTCOME trial (Empagliflozin Cardiovascular Outcome Event Trial in Type 2 Diabetes Mellitus Patients) in patients with type 2 diabetes mellitus and high cardiovascular risk, empagliflozin added to standard of care significantly reduced the risk of the primary outcome 3-point major adverse cardiovascular events (the composite of cardiovascular death, nonfatal myocardial infarction, or nonfatal stroke; hazard ratio [HR], 0.86; 95.02% confidence interval [CI], 0.74–0.99; *P*=0.04).^10^ This was driven primarily by a reduction in the risk of cardiovascular death (HR, 0.62; 95% CI, 0.49–0.77; *P*<0.001). There were no significant differences between empagliflozin and placebo in the risk of myocardial infarction (HR, 0.87; 95% CI, 0.70–1.09; *P*=0.23) or stroke (HR, 1.18; 95% CI, 0.89–1.56; *P*=0.26).^10^ Given the importance of stroke prevention in patients with type 2 diabetes mellitus and the numeric difference in the proportion of patients with stroke events between the empagliflozin and placebo groups in the EMPA-REG OUTCOME trial, we performed a comprehensive analysis of cerebrovascular events in EMPA-REG OUTCOME, including sensitivity and subgroup analyses.

## Methods

### Study Design

The design of EMPA-REG OUTCOME has been described.^10,11^ Briefly, the study population comprised patients with type 2 diabetes mellitus, established cardiovascular disease, and estimated glomerular filtration rate (MDRD [Modification of Diet in Renal Disease] equation) >30 mL min^−1^ 1.73 m^−2^. Patients were randomized 1:1:1 to receive empagliflozin 10 mg, empagliflozin 25 mg, or placebo in addition to standard of care. Throughout the trial (or after week 12 for glucose-lowering medication), investigators were encouraged to treat cardiovascular risk factors to achieve optimal standard of care according to local guidelines. Patients were asked to attend the clinic at prespecified times, including a follow-up visit 30 days after the end of treatment. The trial was to continue until ≥691 patients had experienced an adjudicated event included in the primary outcome. Patients who prematurely discontinued study medication continued to be followed for ascertainment of cardiovascular outcomes, adverse events, and vital status.

The trial was conducted in accordance with the principles of the Declaration of Helsinki and the International Conference on Harmonization Good Clinical Practice guidelines and was approved by local authorities. An independent ethics committee or institutional review board approved the clinical protocol at every participating center. All patients provided written informed consent before study entry.

### Outcomes

Definitions of the major clinical outcomes in EMPA-REG OUTCOME have been described.^10^ The definitions of transient ischemic attack (TIA) and stroke are provided in the online-only Data Supplement. Cardiovascular outcome events and deaths were prospectively adjudicated by 2 Clinical Events Committees (for cardiac and neurological events). We assessed time to first stroke (fatal or nonfatal), time to fatal stroke, time to first nonfatal stroke, time to first nonfatal disabling stroke (defined as adjudicated nonfatal stroke with investigator-reported seriousness criterion of persistent or significant disability/incapacity; stroke disability scores were not used), recurrent stroke during the trial, time to first nonfatal disabling stroke or fatal stroke, time to first cardiovascular death or nonfatal stroke, time to first TIA, and time to first nonfatal or fatal stroke or TIA. Ischemic stroke was classified post hoc according to TOAST criteria (Trial of Org 10 172 in Acute Stroke Treatment)^12^ by the neurological Clinical Events Committee. The Clinical Events Committee charter for classification of ischemic stroke is provided in the online-only Data Supplement.

### Analyses

It was prespecified that analyses would compare the pooled empagliflozin dose groups versus placebo. Outcomes were analyzed using a modified intent-to-treat approach in the treated set (patients treated with ≥1 dose of study drug), using the time to first stroke event irrespective of whether another outcome event had occurred. Data for patients who did not have an event were censored on the last day they were known to be free of the outcome. Sensitivity analyses of fatal or nonfatal stroke were performed based on events that occurred during treatment or ≤90, ≤30, or ≤7 days after a patient’s last intake of study drug (treated set plus 90 days, treated set plus 30 days, and treated set plus 7 days) and based on events that occurred during treatment or ≤30 days after a patient’s last intake of study drug in patients who received ≥30 days of study medication (cumulative; on-treatment set). A sensitivity analysis of TIA was performed on the treated set plus 90 days. Analyses were based on a Cox proportional hazards model, with treatment, age, sex, baseline body mass index, baseline HbA1c, baseline estimated glomerular filtration rate, and region as factors. Subgroup analyses included a subgroup factor and a treatment-by-subgroup factor interaction as additional effects. All analyses were performed at a nominal level of α=0.05 2-sided without adjustment for multiplicity. Cumulative incidence function estimates were corrected for death as a competing risk. Because of the declining numbers of patients at risk, cumulative incidence plots have been truncated at 48 months.

The percentages of patients with recurrent stroke were analyzed descriptively in the treated set. In addition, analyses were conducted of the percentages of patients with stroke in patients with maximum decreases from baseline in systolic blood pressure ≥30 and <30 mm Hg, with maximum increases from baseline in hematocrit ≥90th and <90th percentiles, in patients who had an event consistent with volume depletion (based on 8 preferred terms in the Medical Dictionary for Regulatory Activities) and in patients who had an atrial fibrillation event (based on the Medical Dictionary for Regulatory Activities preferred term). Changes from baseline in systolic blood pressure and hematocrit at the last value on treatment and at follow-up were analyzed descriptively.

Prespecified analyses were the modified intent-to-treat analyses and analyses in the on-treatment set for time to first fatal or nonfatal stroke, nonfatal stroke and TIA, and the assessment of recurrent strokes. Other analyses were post hoc.

## Results

### Study Population

A total of 7020 patients at 590 sites in 42 countries received ≥1 dose of study drug. Baseline characteristics of the study population have been described.^10^ Briefly, mean (SD) age was 63.1 (8.6) years, mean (SD) body mass index was 30.6 (5.3) kg/m^2^, 71.5% were male, 25.9% had estimated glomerular filtration rate <60 mL min^−1^ 1.73 m^−2^, 39.6% had microalbuminuria or macroalbuminuria, 38.7% had systolic blood pressure ≥140 mm Hg or diastolic blood pressure ≥90 mm Hg, 23.3% had a history of stroke, 5.5% of patients had atrial fibrillation, 89.1% were taking anticoagulant or antiplatelet therapies, and 82.7% were taking acetylsalicylic acid.^10,13^ In total, 97% of patients completed the study, and 25% prematurely discontinued study drug. The median duration of treatment was 2.6 years, and the median observation time was 3.1 years. Vital status was available for 99% of patients.

### Stroke and TIA

During the trial, 3.0% (69/2333) of patients in the placebo group and 3.5% (164/4687) of patients in the empagliflozin group had ≥1 adjudicated fatal or nonfatal stroke. Ischemic stroke was reported in 2.7% and 3.2% of patients in the placebo and empagliflozin groups and hemorrhagic stroke in 0.3% and 0.2% of patients in these groups, respectively. A further 0.1% of patients in each group had a stroke for which the type was not assessable. There was no marked imbalance between the placebo and empagliflozin groups in any specific type of ischemic stroke. Cardioembolism was the most common type of ischemic stroke that could be determined (Table I in the online-only Data Supplement).

**Figure 1.**
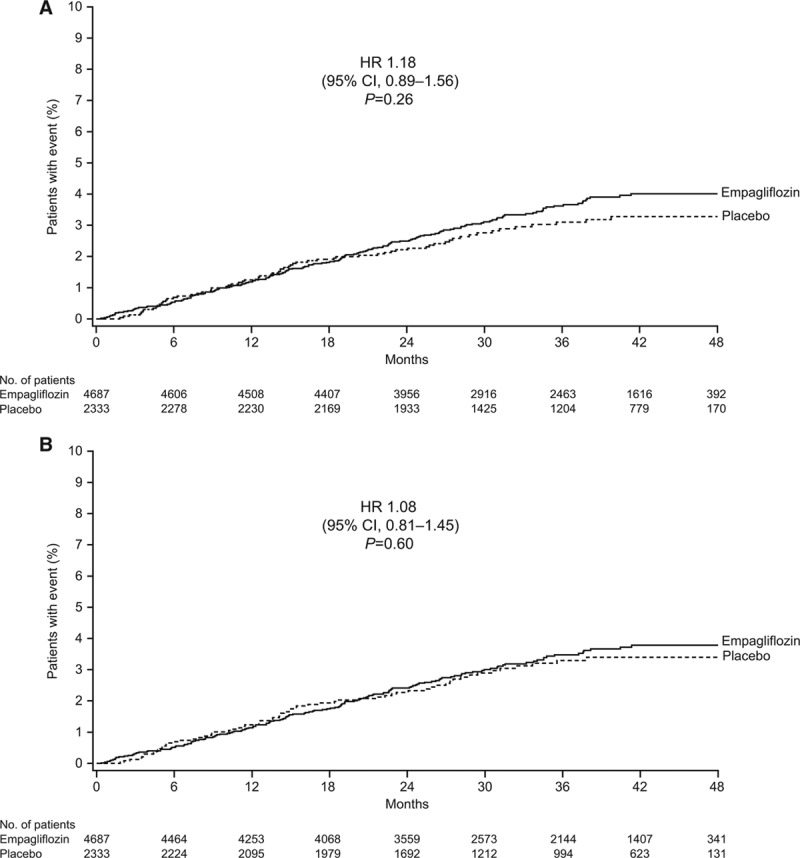
Time to first fatal or nonfatal stroke. **A**, Modified intent-to-treat analyses in the treated set; events observed from randomization to the end of the study in treated set (patients treated with ≥1 dose of study drug). **B**, Sensitivity analysis in treated set plus 90 days; events observed during treatment or ≤90 days after a patient’s last intake of trial medication in treated set (patients treated with ≥1 dose of study drug). Cumulative incidence function. Hazard ratios (HR) are based on Cox regression analyses. CI indicates confidence interval.

In the prespecified modified intent-to-treat analysis of time to first stroke, there was no significant difference between empagliflozin and placebo in the occurrence of stroke (HR, 1.18; 95% CI, 0.89–1.56; *P*=0.26).^10^ The cumulative incidence of time to first stroke is shown in Figure 1A. In sensitivity analyses based on events that occurred during treatment or ≤90, ≤30, or ≤7 days after the last dose of study drug, there was no significant difference in the occurrence of stroke between empagliflozin and placebo, and the HR moved toward unity compared with the modified intent-to-treat analysis (Figure I in the online-only Data Supplement). The numeric difference in the proportion of patients with stroke between the empagliflozin and placebo groups was largely driven by events that occurred >90 days after a patient’s last intake of trial medication (Figure 1B; Figure I in the online-only Data Supplement). Three patients treated with placebo and 18 patients treated with empagliflozin experienced their first stroke >90 days after the last intake of trial medication (of whom 1 patient in the placebo group and 11 patients in the empagliflozin group experienced their first stroke >1 year after the last intake of trial medication).

**Figure 2.**
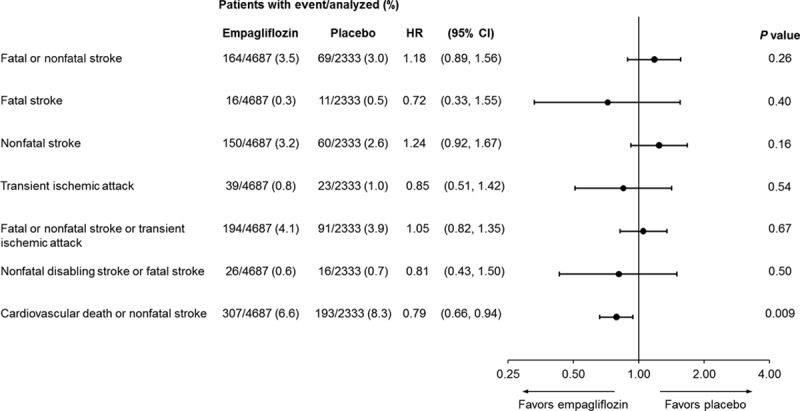
Time to first stroke, transient ischemic attack, and composite outcomes in modified intent-to-treat analyses. Cox regression analyses. Events from randomization to the end of the study in treated set (patients treated with ≥1 dose of study drug). Analyses were prespecified for time to first fatal or nonfatal stroke, time to first nonfatal stroke, and time to first transient ischemic attack. CI indicates confidence interval; and HR, hazard ratio.

The proportion of patients with recurrent stroke during the trial was similar between the empagliflozin and placebo groups (13 [0.3%] and 8 [0.3%], respectively; Table II in the online-only Data Supplement). Nonfatal disabling stroke (based on investigator-reported seriousness criterion [not stroke disability scores]) was reported in 10 patients (0.2%) on empagliflozin and 6 patients (0.3%) on placebo (HR, 0.82; 95% CI, 0.30–2.26; *P*=0.70). Fatal stroke was reported in similar proportions of patients in the empagliflozin and placebo groups (0.3% and 0.5%, respectively; HR, 0.72; 95% CI, 0.33–1.55; *P*=0.40) as was the composite of nonfatal disabling stroke or fatal stroke (0.6% and 0.7%, respectively; HR, 0.81; 95% CI, 0.43–1.50; *P*=0.50; Figure 2).

As empagliflozin reduced the risk of cardiovascular death by 38%,^10^ the composite outcome of cardiovascular death or nonfatal stroke was analyzed to account for cardiovascular death as a competing risk. Empagliflozin significantly reduced the risk of this composite outcome (HR, 0.79; 95% CI, 0.66–0.94; *P*=0.009; Figure 2).

There was no significant difference in the risk of TIA (HR, 0.85; 95% CI, 0.51–1.42; *P*=0.54) or the composite of stroke or TIA (HR, 1.05; 95% CI, 0.82–1.35; *P*=0.87) with empagliflozin versus placebo (Figure 2; Figure II in the online-only Data Supplement).

### Subgroup Analyses

**Figure 3.**
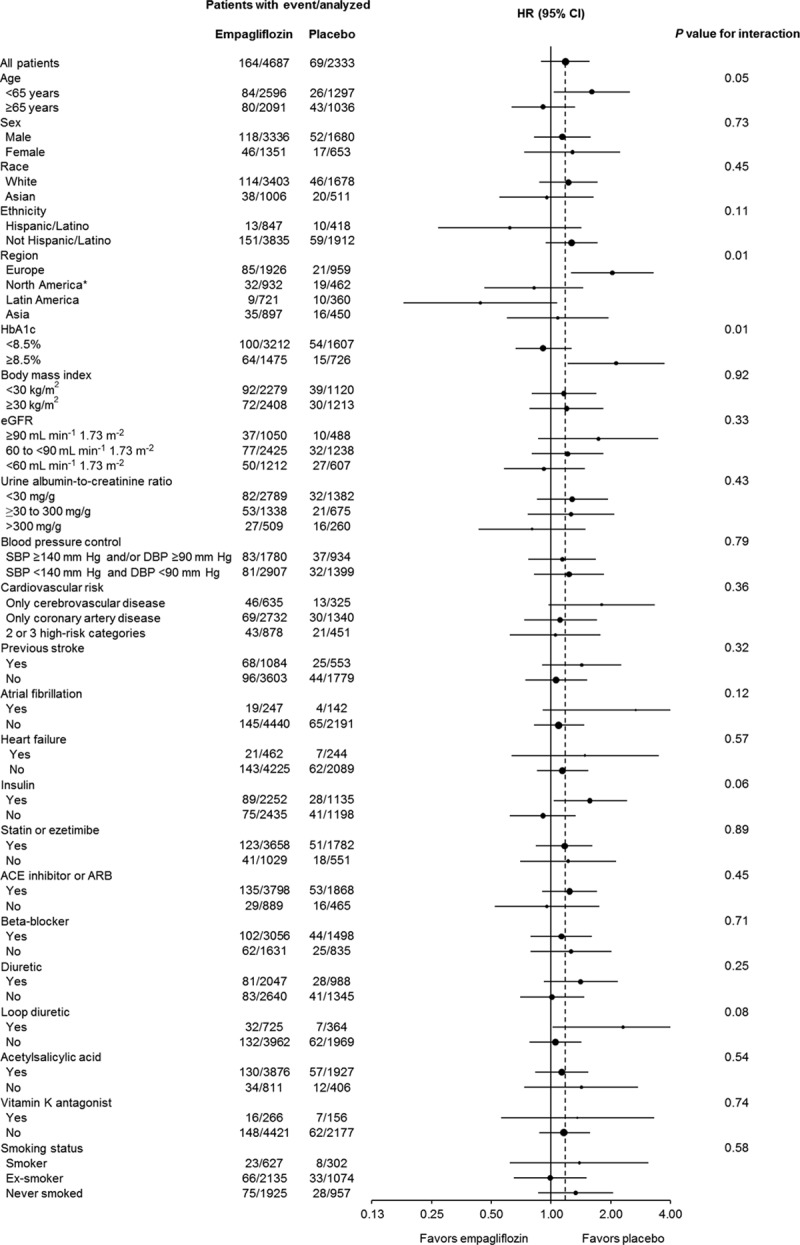
Time to first stroke in subgroups defined by baseline characteristics. Post hoc Cox regression analyses. Events of fatal or nonfatal stroke observed from randomization to end of study in treated set (patients treated with ≥1 dose of study drug). Race: Black and Other not included in Cox regression as <14 patients with an event in these subgroups. Region: Africa not included in Cox regression as <14 patients with an event in this subgroup. Cardiovascular risk: no high cardiovascular risk not included in Cox regression as <14 patients with an event in this subgroup. Heart failure at baseline was based on narrow standardized Medical Dictionary for Regulatory Activities query cardiac failure. *P* value is for homogeneity of the treatment group difference among subgroups (test for group by covariate interaction) with no adjustment for multiple tests. *P*=0.054 for age. The size of the oval is proportional to the number of patients in the subgroup. ACE indicates angiotensin-converting enzyme; ARB, angiotensin receptor blocker; CI, confidence interval; DBP, diastolic blood pressure; eGFR, estimated glomerular filtration rate (according to Modification of Diet in Renal Disease formula); HbA1c, glycated hemoglobin; HR, hazard ratio; and SBP, systolic blood pressure. *Plus Australia and New Zealand.

In exploratory analyses of time to first stroke in >30 prespecified subgroups by baseline characteristics, analyses by region and HbA1c showed nominal heterogeneity at *P*<0.05 (with no adjustment for multiple tests; Figure 3; Table III in the online-only Data Supplement). Compared with the total population, the HR for stroke in patients in Europe was higher (2.04; 95% CI, 1.26–3.29; *P* value for interaction of treatment and region: 0.01; Table III in the online-only Data Supplement). Baseline characteristics, including background medications, were similar between treatment groups within a given region (Table IV in the online-only Data Supplement). However, there were small differences in baseline characteristics between patients in Europe and North America (Table IV in the online-only Data Supplement), including a greater proportion of patients in Europe with a history of stroke (Europe: 29.9% placebo, 25.7% empagliflozin; North America: 14.9% placebo, 18.1% empagliflozin). Despite this, patients treated with placebo had a markedly lower stroke rate in Europe than North America (7.8/1000 versus 15.2/1000 patient-years). This pattern was not observed in patients treated with empagliflozin (stroke event rates were 15.7/1000 and 12.3/1000 patient-years in Europe and North America, respectively). In contrast to the HRs for stroke, the HR for TIA in European patients was <1 (0.61; 95% CI, 0.27–1.35) and in North American patients was 1.22 (95% CI, 0.53–2.78; *P* value for interaction of treatment and region: 0.24).

Compared with the total population, the HR for stroke in patients with baseline HbA1c ≥8.5% was higher (*P* value for interaction: 0.01; Table III in the online-only Data Supplement). An analysis by baseline HbA1c deciles showed no significant treatment-by-subgroup interaction (Figure III in the online-only Data Supplement).

Subgroup analysis by risk factors for stroke such as previous stroke, atrial fibrillation, smoking, and hypertension at baseline showed no statistically significant interaction with treatment for risk of stroke (Figure 3; Table III in the online-only Data Supplement).

### Changes in Systolic Blood Pressure, Changes in Hematocrit, and Events Consistent With Volume Depletion or Atrial Fibrillation in Relation to Stroke

Because treatment with empagliflozin is associated with reductions in systolic blood pressure and small increases in hematocrit, we assessed changes in systolic blood pressure and hematocrit in relation to stroke. We assessed the occurrence of stroke in patients who did and did not have events consistent with volume depletion or atrial fibrillation.

Systolic blood pressure decreased in patients treated with empagliflozin (mean change from baseline to last value on treatment: −3.3 [SE, 0.3] mm Hg) but had returned to its baseline level at follow-up (30 days after end of treatment; Table). Patients with the largest decreases from baseline in systolic blood pressure (≥30 mm Hg) did not have an increased risk of stroke compared with other patients (Table V in the online-only Data Supplement).

**Table.**
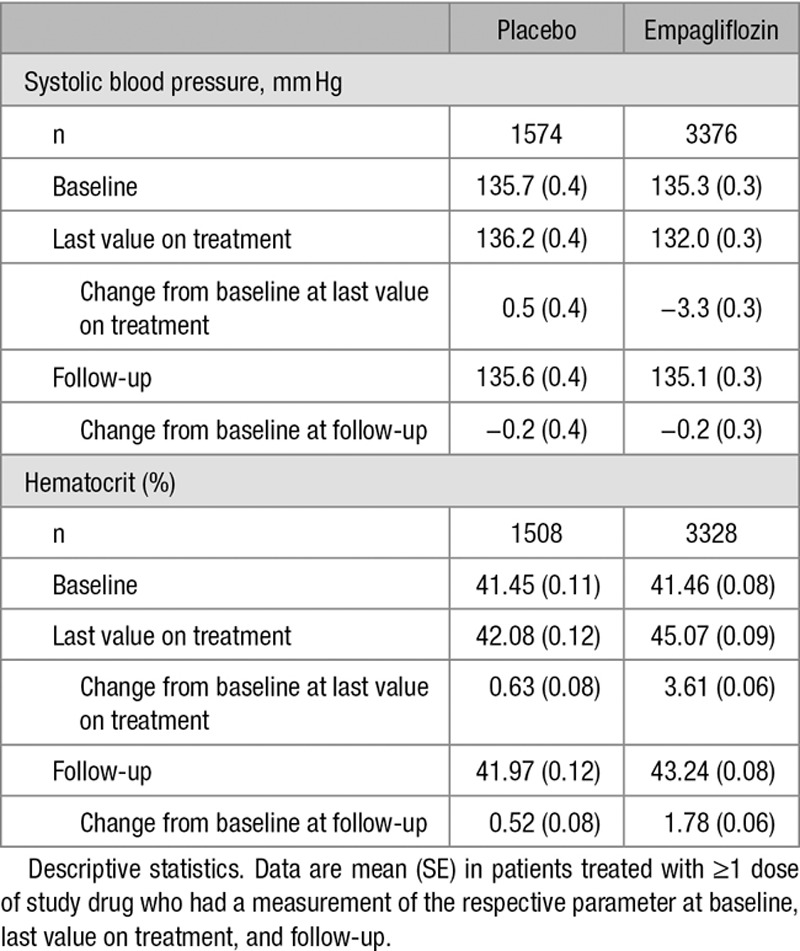
Systolic Blood Pressure and Hematocrit at Baseline, Last Value on Treatment, and at Follow-Up

In the empagliflozin group, hematocrit increased during treatment (mean change from baseline to last value on treatment: 3.61% [SE, 0.06]), but decreased toward baseline at follow-up (30 days after end of treatment; Table). Patients with the largest increases from baseline in hematocrit (increases ≥90th percentile, which corresponded to a change in hematocrit of 9 percentage points) did not have an increased risk of stroke compared with patients not meeting this threshold (Table V in the online-only Data Supplement).

The proportion of patients with a stroke event was not higher in those who did versus did not have an event consistent with volume depletion in placebo or empagliflozin groups (Table VI in the online-only Data Supplement).

The risk of stroke was comparable between patients with and without atrial fibrillation at baseline or during the study in the placebo and empagliflozin groups (Figure 3; Tables III and VII in the online-only Data Supplement).

## Discussion

In the EMPA-REG OUTCOME trial in patients with type 2 diabetes mellitus and high cardiovascular risk, empagliflozin added to standard of care significantly reduced the risk of the primary outcome of 3-point major adverse cardiovascular events by reducing the risk of cardiovascular death. There was no significant difference in the occurrence of stroke between empagliflozin and placebo in the prespecified modified intent-to-treat analysis, but there was a numeric difference between treatment groups. In these new analyses, we show that this numeric difference was driven by nonfatal ischemic stroke, with no isolated increase in any subtype of ischemic stroke, and that there was no significant difference between empagliflozin and placebo in the risk of stroke in on-treatment sensitivity analyses or in the risk of recurrent, fatal, or nonfatal disabling strokes, or TIA, which has similar pathophysiological mechanisms as stroke. Further sensitivity analyses demonstrated that the numeric difference in the proportion of patients with stroke between empagliflozin and placebo in the modified intent-to-treat analysis was primarily because of events that occurred >90 days after the last intake of study drug. In this context, it is important to note that measures of the hemodynamic effects of empagliflozin, specifically systolic blood pressure and hematocrit, returned to near baseline levels within 30 days after the last intake, making a causal association with empagliflozin unlikely and making it unlikely that there is an increased risk of stroke after empagliflozin is stopped.

In subgroup analyses of time to first stroke, analyses by region and HbA1c showed nominal heterogeneity. The increased HR for stroke with empagliflozin compared with placebo in patients in Europe compared with the total population could not be explained by differences in baseline characteristics between regions. Given the large number of subgroup factors and tests conducted, the differences in HR between Europe and other regions, and between patients with HbA1c ≥8.5% and <8.5% at baseline, are within the realm of chance variation. Analyses of time to first stroke in subgroups by other baseline characteristics, including factors associated with risk for stroke such as atrial fibrillation, smoking, previous stroke, and hypertension,^14–17^ showed no statistically significant interaction with treatment for the risk of stroke.

The risk of experiencing a stroke was comparable between patients with and without atrial fibrillation at baseline or during the study. A slightly lower proportion of patients treated with empagliflozin than placebo had anticoagulants introduced postbaseline,^10^ and it cannot be excluded that this could have contributed to the numeric difference in stroke.

Treatment with empagliflozin is associated with hemoconcentration, as shown by increases in hematocrit, and with reductions in systolic blood pressure.^10^ Concerns have been raised that elevated hematocrit and hypotension may be associated with an increased risk of stroke caused by sludging and hypoperfusion, respectively. In a meta-analysis of observational studies of patients with and without diabetes mellitus, orthostatic hypotension was associated with an increased risk of cardiovascular events, including stroke.^18^ In EMPA-REG OUTCOME, mean baseline hematocrit in the empagliflozin group was 41.4%, mean baseline systolic blood pressure was 135 mm Hg, and patients with the largest increases in hematocrit and the largest decreases in systolic blood pressure did not have an increased risk of stroke. The proportion of patients with a stroke event was consistent between patients who did and did not have an event consistent with volume depletion in both treatment groups.

A reduction in the risk of stroke was observed with intensive blood pressure lowering versus standard therapy in the ACCORD study (Action to Control Cardiovascular Risk in Diabetes), but this occurred after a mean follow-up of 4.7 years despite a large difference in systolic blood pressure (14.2 mm Hg) after 1 year.^19^ Thus, the lack of a risk reduction for stroke with empagliflozin in EMPA-REG OUTCOME may have been expected given the modest reduction in systolic blood pressure provided by empagliflozin over a median treatment time of 2.6 years and from a baseline of 135 mm Hg with 95% of patients taking antihypertensive therapy at baseline. The risk of stroke in the EMPA-REG OUTCOME trial was similar between patients with controlled (systolic blood pressure <140 mm Hg and diastolic blood pressure <90 mm Hg) versus uncontrolled blood pressure at baseline.

Limitations of these analyses include that the results cannot be extrapolated beyond the treatment duration or observation time of the trial or to patient populations with other clinical characteristics.

In conclusion, in patients with type 2 diabetes mellitus and high cardiovascular risk in the EMPA-REG OUTCOME trial, empagliflozin, when compared with placebo, was not associated with either a reduction or an increase in the risk of cerebrovascular events.

## Acknowledgments

Medical writing assistance, supported financially by Boehringer Ingelheim, was provided by Elizabeth Ng and Wendy Morris of FleishmanHillard Fishburn, London, United Kingdom, during the preparation of this article. The authors are fully responsible for all content and editorial decisions, were involved at all stages of article development, and have approved the final version.

## Sources of Funding

This trial was funded by the Boehringer Ingelheim and Eli Lilly and Company Diabetes Alliance.

## Disclosures

Dr Zinman has received personal fees from AstraZeneca, Boehringer Ingelheim, Eli Lilly and Company, Janssen, Merck & Co, Novo Nordisk, Sanofi, and Takeda and has received grants from Boehringer Ingelheim, Novo Nordisk, and Merck & Co. Dr Inzucchi has received personal fees from Alere, AstraZeneca, Daiichi-Sankyo, Janssen, Intarcia, Merck & Co, Novo Nordisk, Poxel, Sanofi, Regeron, Lexicon, vTv Pharmaceuticals, and Eli Lilly and Company; received personal fees and nonfinancial support from Boehringer Ingelheim; received nonfinancial support from Takeda; and has received grants from the National Institute of Diabetes and Digestive and Kidney Diseases and the National Institute of Neurological Disorders and Stroke. Dr Lachin has received personal fees from Boehringer Ingelheim, Merck & Co, Gilead Sciences, Janssen, Novartis, and AstraZeneca. Dr Wanner has received personal fees from Boehringer Ingelheim, Janssen, and Novo Nordisk; and has received grants from Boehringer Ingelheim and the European Foundation for the Study of Diabetes. Dr Fitchett has received personal fees from Boehringer Ingelheim, Novo Nordisk, AstraZeneca, Sanofi, and Merck & Co. Dr Albers reports personal fees from AstraZeneca, Codman, Covidien, iSchemaView, Genentech, Johnson & Johnson, and Lundbeck; has received grants from Lundbeck; and has a patent on Automated arterial input function detection issued. Dr Diener has received honoraria for participation in clinical trials, contribution to advisory boards or presentations from Abbott, Allergan, AstraZeneca, Bayer Vital, Bristol-Myers Squibb, Boehringer Ingelheim, CoAxia, Corimmun, Covidien, Daiichi-Sankyo, D-Pharm, Fresenius, GlaxoSmithKline, Janssen-Cilag, Johnson & Johnson, Knoll, Lilly, Merck Sharp & Dohme, Medtronic, MindFrame, Neurobiological Technologies, Novartis, Novo Nordisk, Paion, Parke-Davis, Pfizer, Sanofi-Aventis, Schering-Plough, Servier, Solvay, St. Jude, Sygnis, Talecris, Thrombogenics, WebMD Global, Wyeth, and Yamanouchi, and has received financial support for research projects from AstraZeneca, GlaxoSmithKline, Boehringer Ingelheim, Lundbeck, Novartis, Janssen-Cilag, Sanofi-Aventis, Sygnis, and Talecris. The Department of Neurology at the University Duisburg-Essen has received research grants from the German Research Council, German Ministry of Education and Research, European Union, the National Institutes of Health, Bertelsmann Foundation, and Heinz-Nixdorf Foundation. Dr Diener served as editor of Aktuelle Neurologie, Arzneimitteltherapie, Kopfschmerznews, Stroke News, as coeditor of Cephalalgia, and is on the editorial board of Lancet Neurology, Stroke, European Neurology, and Cerebrovascular Disorders. Dr Diener chairs the treatment guidelines committee of the German Society of Neurology and contributed to the European Heart Rhythm Association and the European Society of Cardiology guidelines for the treatment of atrial fibrillation. Dr Kohler, M. Mattheus, Dr Johansen, Dr Woerle, and Dr Broedl are employees of Boehringer Ingelheim.

## Supplementary Material
