# Supplementary material for: Empagliflozin and Cerebrovascular Events in Patients With Type 2 Diabetes Mellitus at High Cardiovascular Risk
Source: Stroke. 2017 Apr 24;48(5):1218–25. doi: 10.1161/STROKEAHA.116.015756 (PMC5404404; doi:10.1161/STROKEAHA.116.015756)
Supplement: Supplementary file 1 [file str-48-1218-s001.pdf]

SUPPLEMENTAL MATERIAL

Supplementary Table I. Type of ischemic stroke according to TOAST classification.[1] Post-hoc analysis.

N (%)	**Placebo (n=2333)**	**Empagliflozin (n=4687)**
Ischemic stroke	62 (2.7)	149 (3.2)
Cardioembolism	22 (0.9)	49 (1.0)
Large-artery atherosclerosis	4 (0.2)	14 (0.3)
Small-vessel occlusion (lacune)	5 (0.2)	11 (0.2)
Other determined etiology	0	2 (<0.1)
Undetermined etiology	32 (1.4)	74 (1.6)

Treated set (patients treated with ≥1 dose of study drug).

Supplementary Table II. Number of stroke events during the trial. Pre-specified analysis.

N (%)	**Placebo (n=2333)**	**Empagliflozin (n=4687)**
Fatal or non-fatal stroke (≥1 event)	69 (3.0)	164 (3.5)
1 event	61 (2.6)	151 (3.2)
Non-fatal	52 (2.2)	137 (2.9)
Fatal	9 (0.4)	14 (0.3)
2 events	6 (0.3)	11 (0.2)
1st non-fatal<br>2nd non-fatal	5 (0.2)	9 (0.2)
1st non-fatal<br>2nd fatal	1 (<0.1)	2 (<0.1)
3 events	1 (<0.1)	2 (<0.1)
1st non-fatal<br>2nd non-fatal<br>3rd non-fatal	1 (<0.1)	2 (<0.1)
4 events	1 (<0.1)	0
1st non-fatal<br>2nd non-fatal<br>3rd non-fatal<br>4th fatal	1 (<0.1)	0

Treated set (patients treated with ≥1 dose of study drug).

Supplementary Table III. Time to first stroke in subgroups defined by baseline characteristics. Post-hoc Cox regression analyses.

	Placebo		Empagliflozin		HR (95% CI)	p-value for interaction
	n (%)	Rate/1000 patient-years	n (%)	Rate/1000 patient-years		
All patients	69 (3.0)	10.5	164 (3.5)	12.3	1.18 (0.89, 1.56)	–
Age						0.05
<65 years	26 (2.0)	7.0	84 (3.2)	11.2	1.60 (1.03, 2.49)	–
≥65 years	43 (4.2)	14.9	80 (3.8)	13.5	0.91 (0.63, 1.32)	–
Sex						0.73
Male	52 (3.1)	10.9	118 (3.5)	12.3	1.14 (0.82, 1.58)	–
Female	17 (2.6)	9.3	46 (3.4)	12.1	1.28 (0.73, 2.23)	–
Race						0.45
White	46 (2.7)	9.8	114 (3.3)	11.8	1.22 (0.87, 1.72)	–
Asian	20 (3.9)	13.2	38 (3.8)	12.6	0.95 (0.55, 1.64)	–
Black/African American*	2 (1.7)	6.0	10 (4.2)	15.7	–	–
Other*	1 (4.2)	18.0	2 (5.0)	19.2	–	–
Ethnicity						0.11
Hispanic/Latino	10 (2.4)	8.7	13 (1.5)	5.4	0.62 (0.27, 1.42)	–
Not Hispanic/Latino	59 (3.1)	10.9	151 (3.9)	13.8	1.27 (0.94, 1.72)	–
Region						0.01
Europe	21 (2.2)	7.8	85 (4.4)	15.7	2.04 (1.26, 3.29)	–
North America (plus Australia and New Zealand)	19 (4.1)	15.2	32 (3.4)	12.3	0.82 (0.46, 1.45)	–
Asia	16 (3.6)	12.0	35 (3.9)	13.0	1.08 (0.60, 1.95)	–
Latin America	10 (2.8)	9.9	9 (1.2)	4.4	0.44 (0.18, 1.07)	–
Africa*	3 (2.9)	10.5	3 (1.4)	5.0	–	–
Time since diagnosis of type 2 diabetes						0.84
≤5 years	11 (2.6)	9.3	22 (2.6)	9.2	0.97 (0.47, 1.99)	–
>5–10 years	13 (2.3)	8.0	33 (2.8)	9.7	1.26 (0.66, 2.40)	–
>10 years	45 (3.4)	12.0	109 (4.1)	14.4	1.20 (0.85, 1.70)	–
HbA1c						0.01
<8.5%	54 (3.4)	12.0	100 (3.1)	10.9	0.91 (0.66, 1.27)	–
≥8.5%	15 (2.1)	7.2	64 (4.3)	15.2	2.13 (1.21, 3.74)	–
Body mass index						0.92
<30 kg/m^2	39 (3.5)	12.2	92 (4.0)	14.0	1.16 (0.80, 1.69)	–

≥30 kg/m^2	30 (2.5)	8.9	72 (3.0)	10.5	1.20 (0.78, 1.83)	–
eGFR						0.33
≥90 mL/min/1.73m^2	10 (2.0)	7.2	37 (3.5)	12.5	1.73 (0.86, 3.47)	–
60 to <90 mL/min/1.73m^2	32 (2.6)	9.1	77 (3.2)	11.0	1.21 (0.80, 1.83)	–
<60 mL/min/1.73m^2	27 (4.4)	16.1	50 (4.1)	14.6	0.92 (0.58, 1.48)	–
Urine albumin-to-creatinine ratio						0.43
<30 mg/g	32 (2.3)	8.0	82 (2.9)	10.2	1.28 (0.85, 1.93)	–
≥30 to 300 mg/g	21 (3.1)	11.1	53 (4.0)	14.0	1.26 (0.76, 2.08)	–
>300 mg/g	16 (6.2)	23.8	27 (5.3)	19.1	0.80 (0.43, 1.49)	–
Blood pressure control						0.79
SBP ≥140 mmHg and/or DBP ≥90 mmHg	37 (4.0)	14.2	83 (4.7)	16.5	1.14 (0.77, 1.68)	–
SBP <140 mmHg and DBP <90 mmHg	32 (2.3)	8.0	81 (2.8)	9.7	1.23 (0.82, 1.85)	–
Cardiovascular risk						0.36
Only cerebrovascular disease	13 (4.0)	14.5	46 (7.2)	26.1	1.80 (0.97, 3.34)	–
Only coronary artery disease	30 (2.2)	7.8	69 (2.5)	8.6	1.11 (0.73, 1.71)	–
Only peripheral artery disease	5 (2.6)	9.6	6 (1.5)	5.4	–	–
2 or 3 high-risk categories	21 (4.7)	17.2	43 (4.9)	17.6	1.05 (0.62, 1.78)	–
Previous stroke						0.32
Yes	25 (4.5)	16.6	68 (6.3)	22.7	1.43 (0.90, 2.26)	–
No	44 (2.5)	8.7	96 (2.7)	9.2	1.06 (0.74, 1.52)	–
Atrial fibrillation						0.12
Yes	4 (2.8)	10.8	19 (7.7)	29.5	2.67 (0.91, 7.86)	–
No	65 (3.0)	10.5	145 (3.3)	11.4	1.09 (0.82, 1.47)	–
Heart failure						0.57
Yes	7 (2.9)	11.5	21 (4.5)	17.4	1.48 (0.63, 3.49)	–
No	62 (3.0)	10.4	143 (3.4)	11.8	1.14 (0.85, 1.54)	–
Hypertension						–
Yes	66 (3.1)	10.9	160 (3.8)	13.2	–	–
No*	3 (1.7)	5.5	4 (1.0)	3.2	–	–
Smoking status						0.58
Never smoked	28 (2.9)	10.3	75 (3.9)	13.7	1.33 (0.86, 2.06)	–
Ex-smoker	33 (3.1)	10.8	66 (3.1)	10.7	0.99 (0.65, 1.51)	–
Smoker	8 (2.6)	9.8	23 (3.7)	13.3	1.39 (0.62, 3.10)	–

Metformin						0.60
Yes	48 (2.8)	9.7	117 (3.4)	11.8	1.23 (0.88, 1.73)	–
No	21 (3.5)	13.0	47 (3.8)	13.6	1.05 (0.63, 1.75)	–
Sulfonylurea						0.54
Yes	25 (2.5)	8.8	66 (3.3)	11.3	1.31 (0.83, 2.08)	–
No	44 (3.3)	11.8	98 (3.7)	13.0	1.10 (0.77, 1.57)	–
Insulin						0.06
Yes	28 (2.5)	8.9	89 (4.0)	14.1	1.57 (1.03, 2.41)	–
No	41 (3.4)	11.9	75 (3.1)	10.6	0.91 (0.62, 1.33)	–
Thiazolidinedione						–
Yes*	2 (2.0)	6.3	6 (3.0)	9.8	–	–
No	67 (3.0)	10.7	158 (3.5)	12.4	–	–
DPP-4 inhibitor						0.12
Yes	8 (3.0)	10.8	31 (5.9)	22.1	2.06 (0.94, 4.48)	–
No	61 (3.0)	10.4	133 (3.2)	11.1	1.07 (0.79, 1.45)	–
Statin or ezetimibe						0.89
Yes	51 (2.9)	10.1	123 (3.4)	11.7	1.17 (0.84, 1.62)	–
No	18 (3.3)	11.7	41 (4.0)	14.2	1.22 (0.70, 2.13)	–
Antihypertensive therapy						–
Yes	65 (2.9)	10.4	156 (3.5)	12.3	–	–
No*	4 (3.6)	12.4	8 (3.3)	12.0	–	–
ACE inhibitor or ARB						0.45
Yes	53 (2.8)	10.1	135 (3.6)	12.5	1.24 (0.90, 1.71)	–
No	16 (3.4)	12.2	29 (3.3)	11.4	0.95 (0.52, 1.75)	–
Calcium channel blocker						0.71
Yes	26 (3.3)	11.7	56 (3.7)	12.8	1.10 (0.69, 1.75)	–
No	43 (2.8)	9.9	108 (3.4)	12.0	1.23 (0.86, 1.74)	–
Beta-blocker						0.71
Yes	44 (2.9)	10.4	102 (3.3)	11.6	1.13 (0.79, 1.61)	–
No	25 (3.0)	10.6	62 (3.8)	13.4	1.26 (0.79, 2.01)	–
Diuretic						0.25
Yes	28 (2.8)	10.2	81 (4.0)	14.0	1.41 (0.92, 2.17)	–
No	41 (3.0)	10.7	83 (3.1)	10.9	1.01 (0.70, 1.47)	–
Loop diuretic						0.08
Yes	7 (1.9)	7.1	32 (4.4)	16.2	2.30 (1.02, 5.22)	–
No	62 (3.1)	11.1	132 (3.3)	11.6	1.05 (0.78, 1.42)	–
Acetylsalicylic acid						0.54
Yes	57 (3.0)	10.4	130 (3.4)	11.7	1.13 (0.83, 1.54)	–
No	12 (3.0)	11.0	34 (4.2)	15.0	1.42 (0.73, 2.74)	–
Vitamin K antagonist						0.74
Yes	7 (4.5)	16.8	16 (6.0)	21.9	1.36 (0.56, 3.32)	–

No	62 (2.8)	10.1	148 (3.3)	11.7	1.16 (0.87, 1.57)	–

*HR and 95% CI were not calculated as <14 patients with an event in this subgroup.

Treated set (patients treated with ≥1 dose of study drug).

p-value is for test of homogeneity of the treatment group difference among subgroups (test for treatment group by covariate interaction) with no adjustment for multiple tests. p=0.0535 for age.

ACE, angiotensin-converting enzyme; ARB, angiotensin receptor blocker; CI, confidence interval; DBP, diastolic blood pressure; HbA1c, glycated haemoglobin; HR, hazard ratio; SBP, systolic blood pressure.

Supplementary Table IV. Baseline characteristics in subgroups of patients by region and treatment.

	Europe		North America (plus Australia and New Zealand)		Latin America		Africa		Asia	
	Placebo (n=959)	Empagliflozin (n=1926)	Placebo (n=462)	Empagliflozin (n=932)	Placebo (n=360)	Empagliflozin (n=721)	Placebo (n=102)	Empagliflozin (n=211)	Placebo (n=450)	Empagliflozin (n=897)
Male	690 (71.9)	1373 (71.3)	359 (77.7)	678 (72.7)	234 (65.0)	469 (65.0)	70 (68.6)	158 (74.9)	327 (72.7)	658 (73.4)
Age, years	64.0 (8.5)	63.8 (8.2)	65.1 (8.4)	64.5 (8.6)	62.6 (8.1)	62.5 (8.4)	59.8 (9.3)	61.3 (8.8)	60.8 (9.4)	61.0 (9.0)
Race										
White	941 (98.1)	1897 (98.5)	392 (84.8)	788 (84.5)	304 (84.4)	611 (84.7)	41 (40.2)	105 (49.8)	0	2 (0.2)
Black/African-American	4 (0.4)	9 (0.5)	44 (9.5)	85 (9.1)	36 (10.0)	77 (10.7)	36 (35.3)	66 (31.3)	0	0
Asian	14 (1.5)	19 (1.0)	17 (3.7)	45 (4.8)	6 (1.7)	8 (1.1)	24 (23.5)	39 (18.5)	450 (100.0)	895 (99.8)
Other	0	1 (0.1)	9 (2.0)	14 (1.5)	14 (3.9)	25 (3.5)	1 (1.0)	1 (0.5)	0	0
BMI, kg/m^2	31.7 (4.7)	31.5 (4.7)	33.3 (5.4)	33.1 (5.4)	29.8 (4.5)	30.0 (5.0)	30.8 (5.1)	30.9 (5.0)	26.3 (3.8)	26.4 (3.9)
Time since diagnosis of T2DM										
<5 years	183 (19.1)	330 (17.1)	46 (9.9)	110 (11.8)	66 (18.3)	128 (17.8)	30 (29.4)	52 (24.6)	98 (21.8)	220 (24.5)
>5 to 10 years	232 (24.2)	518 (26.9)	109 (23.6)	213 (22.9)	75 (20.8)	178 (24.7)	19 (18.6)	52 (24.6)	136 (30.2)	214 (23.9)
>10 years	544 (56.7)	1078 (56.0)	307 (66.5)	609 (65.3)	219 (60.8)	415 (57.6)	53 (52.0)	107 (50.7)	216 (48.0)	463 (51.6)
eGFR, mL/min/1.73m^2	76.0 (20.5)	76.5 (21.1)	66.6 (18.7)	67.1 (18.7)	76.4 (21.0)	76.3 (23.4)	75.9 (24.6)	76.0 (24.2)	74.0 (22.1)	74.4 (21.8)
Any CV risk factor	949 (99.0)	1920 (99.7)	456 (98.7)	925 (99.2)	353 (98.1)	712 (98.8)	102 (100.0)	211 (100.0)	447 (99.3)	889 (99.1)
CAD	679 (70.8)	1408 (73.1)	414 (89.6)	824 (88.4)	241 (66.9)	452 (62.7)	89 (87.3)	178 (84.4)	340 (75.6)	683 (76.1)

Multi-vessel CAD	371 (38.7)	801 (41.6)	300 (64.9)	583 (62.6)	121 (33.6)	203 (28.2)	58 (56.9)	102 (48.3)	250 (55.6)	490 (54.6)
History of MI	476 (49.6)	985 (51.1)	210 (45.5)	429 (46.0)	160 (44.4)	307 (42.6)	56 (54.9)	110 (52.1)	181 (40.2)	359 (40.0)
Coronary artery bypass graft	189 (19.7)	399 (20.7)	201 (43.5)	389 (41.7)	72 (20.0)	154 (21.4)	43 (42.2)	95 (45.0)	58 (12.9)	138 (15.4)
History of stroke	287 (29.9)	495 (25.7)	69 (14.9)	169 (18.1)	70 (19.4)	155 (21.5)	17 (16.7)	31 (14.7)	110 (24.4)	234 (26.1)
Peripheral artery disease	226 (23.6)	450 (23.4)	86 (18.6)	199 (21.4)	101 (28.1)	212 (29.4)	12 (11.8)	38 (18.0)	54 (12.0)	83 (9.3)
Single vessel CAD	111 (11.6)	230 (11.9)	60 (13.0)	122 (13.1)	13 (3.6)	28 (3.9)	5 (4.9)	17 (8.1)	49 (10.9)	101 (11.3)
Atrial fibrillation*	78 (8.1)	129 (6.7)	40 (8.7)	66 (7.1)	13 (3.6)	20 (2.8)	2 (2.0)	10 (4.7)	9 (2.0)	22 (2.5)
Any anti-hypertensive drugs	917 (95.6)	1846 (95.8)	449 (97.2)	902 (96.8)	338 (93.9)	669 (92.8)	101 (99.0)	200 (94.8)	416 (92.4)	829 (92.4)
ACE inhibitors/ARBs	795 (82.9)	1616 (83.9)	377 (81.6)	765 (82.1)	300 (83.3)	594 (82.4)	84 (82.4)	179 (84.8)	312 (69.3)	644 (71.8)
Beta-blockers	629 (65.6)	1326 (68.8)	335 (72.5)	670 (71.9)	209 (58.1)	407 (56.4)	69 (67.6)	133 (63.0)	256 (56.9)	520 (58.0)
Diuretics	461 (48.1)	953 (49.5)	214 (46.3)	445 (47.7)	142 (39.4)	298 (41.3)	56 (54.9)	125 (59.2)	115 (25.6)	226 (25.2)
Calcium channel blockers	354 (36.9)	668 (34.7)	137 (29.7)	251 (26.9)	97 (26.9)	182 (25.2)	31 (30.4)	80 (37.9)	169 (37.6)	348 (38.8)
Mineralocorticoid receptor antagonists	62 (6.5)	140 (7.3)	15 (3.2)	43 (4.6)	36 (10.0)	60 (8.3)	10 (9.8)	12 (5.7)	13 (2.9)	50 (5.6)
Renin inhibitors	15 (1.6)	17 (0.9)	1 (0.2)	5 (0.5)	2 (0.6)	4 (0.6)	0	0	1 (0.2)	1 (0.1)
Other	95 (9.9)	183 (9.5)	54 (11.7)	105 (11.3)	19 (5.3)	28 (3.9)	3 (2.9)	16 (7.6)	20 (4.4)	51 (5.7)
Any lipid-lowering drugs	736 (76.7)	1515 (78.7)	402 (87.0)	833 (89.4)	271 (75.3)	551 (76.4)	98 (96.1)	189 (89.6)	357 (79.3)	732 (81.6)
Statins	705 (73.5)	1448 (75.2)	368 (79.7)	763 (81.9)	255 (70.8)	527 (73.1)	98 (96.1)	188 (89.1)	347 (77.1)	704 (78.5)
Fibrates	64 (6.7)	163 (8.5)	64 (13.9)	126 (13.5)	36 (10.0)	74 (10.3)	3 (2.9)	9 (4.3)	32 (7.1)	59 (6.6)

Ezetimibe	34 (3.5)	72 (3.7)	20 (4.3)	69 (7.4)	13 (3.6)	24 (3.3)	2 (2.0)	2 (0.9)	12 (2.7)	22 (2.5)
Niacin	7 (0.7)	9 (0.5)	23 (5.0)	75 (8.0)	1 (0.3)	3 (0.4)	0	1 (0.5)	4 (0.9)	3 (0.3)
Other	33 (3.4)	82 (4.3)	123 (26.6)	251 (26.9)	3 (0.8)	5 (0.7)	3 (2.9)	3 (1.4)	13 (2.9)	24 (2.7)
Any anti-coagulant/anti-platelet drugs	845 (88.1)	1694 (88.0)	418 (90.5)	847 (90.9)	309 (85.8)	616 (85.4)	100 (98.0)	192 (91.0)	418 (92.9)	813 (90.6)
Acetylsalicylic acid	755 (78.7)	1540 (80.0)	395 (85.5)	807 (86.6)	299 (83.1)	588 (81.6)	96 (94.1)	185 (87.7)	382 (84.9)	756 (84.3)
Vitamin K antagonists	87 (9.1)	148 (7.7)	44 (9.5)	63 (6.8)	10 (2.8)	24 (3.3)	5 (4.9)	11 (5.2)	10 (2.2)	17 (1.9)
Clopidogrel	81 (8.4)	183 (9.5)	18 (3.9)	36 (3.9)	51 (14.2)	108 (15.0)	7 (6.9)	15 (7.1)	92 (20.4)	152 (16.9)
Non-vitamin K antagonist oral anticoagulants†	4 (0.4)	4 (0.2)	7 (1.5)	6 (0.6)	1 (0.3)	0	0	0	1 (0.2)	2 (0.2)

Data are n (%) or mean (SD) in the treated set (patients treated with ≥1 dose of study drug).

*Based on 1 MedDRA preferred term.

†Direct factor Xa inhibitors or direct thrombin inhibitors.

BMI, body mass index; eGFR, estimated glomerular filtration rate; T2DM, type 2 diabetes; CV, cardiovascular; CAD, coronary artery disease; ACE, angiotensin-converting enzyme; ARB, angiotensin receptor blockers; MedDRA, Medical Dictionary for Regulatory Activities.

Supplementary Table V. Time to first fatal or non-fatal stroke by maximum increase from baseline in hematocrit or maximum decrease from baseline in systolic blood pressure based on data prior to or on date of first fatal or non-fatal stroke. Post-hoc analyses.

	**Placebo**	**Empagliflozin**
	**n with event/ n analyzed (%)**	**n with event/ n analyzed (%)**
**Incidence of stroke by maximum increase from baseline in hematocrit before or on date of first stroke (in patients with stroke) or censoring (in patients without stroke)**		
Patients with increase from baseline in hematocrit ≥90th percentile	1/75 (1.3)	11/705 (1.6)
Patients with increase from baseline in hematocrit <90th percentile	65/2211 (2.9)	133/3862 (3.4)
**Incidence of stroke by maximum decrease from baseline in systolic blood pressure before or on date of first stroke (in patients with stroke) or censoring (in patients without stroke)**		
Patients with decrease from baseline in systolic blood pressure of ≥30 mmHg	55/1907 (2.9)	122/3596 (3.4)
Patients with decrease from baseline in systolic blood pressure of <30 mmHg	14/415 (3.4)	39/1049 (3.7)

Treated set (patients treated with ≥1 dose of study drug).
Hematocrit: 90th percentile corresponds to a change from baseline of 9.0%.

Supplementary Table VI. Stroke in patients with events consistent with volume depletion*. Post-hoc analyses.

N (%)	**Patients with an event consistent with volume depletion**		**Patients without an event consistent with volume depletion**	
	Placebo (n=115)	Empagliflozin (n=239)	Placebo (n=2218)	Empagliflozin (n=4448)
First stroke event after first volume depletion event or during study**	3 (2.6)	8 (3.3)	63 (2.8)	152 (3.4)

Treated set (patients treated with ≥1 dose of study drug).

*Based on 8 Medical Dictionary for Regulatory Activities (MedDRA) preferred terms.
**Only the first stroke per patient was considered. For patients with volume depletion, only a stroke event after the first volume depletion event was considered. For patients without volume depletion, the first stroke in the study was considered.

Supplementary Table VII. Stroke in patients with and without atrial fibrillation* Post-hoc analyses.

N (%)	**Patients with an atrial fibrillation event**		**Patients without an atrial fibrillation event**	
	Placebo (n=67)	Empagliflozin (n=161)	Placebo (n=2266)	Empagliflozin (n=4526)
First stroke event after first atrial fibrillation event or during study**	2 (3.0)	6 (3.7)	65 (2.9)	154 (3.4)

Treated set (patients treated with ≥1 dose of study drug).

*Based on the Medical Dictionary for Regulatory Activities (MedDRA) preferred term ‘atrial fibrillation’.

**Only the first stroke per patient was considered. For patients with an atrial fibrillation event, only a stroke event after the first atrial fibrillation event was considered. For patients without an atrial fibrillation event, the first stroke in the study was considered.

Supplementary Figure I. Time to first fatal or non-fatal stroke in modified intent-to-treat and sensitivity analyses. Cox regression analyses.

	Patients with event/analysed (%)				
	Empagliflozin	Placebo	HR	(95% CI)	p-value
**Fatal or non-fatal stroke**					
Modified intent-to-treat analysis*					
Treated set	164/4687 (3.5)	69/2333 (3.0)	1.18	(0.89, 1.56)	0.26
Sensitivity analyses					
Treated set + 90 days†	146/4687 (3.1)	66/2333 (2.8)	1.08	(0.81, 1.45)	0.60
Treated set + 30 days†	143/4687 (3.1)	66/2333 (2.8)	1.06	(0.79, 1.41)	0.71
Treated set + 7 days†	139/4687 (3.0)	62/2333 (2.7)	1.09	(0.81, 1.48)	0.55
On-treatment set‡	141/4607 (3.1)	66/2308 (2.9)	1.04	(0.78, 1.40)	0.78

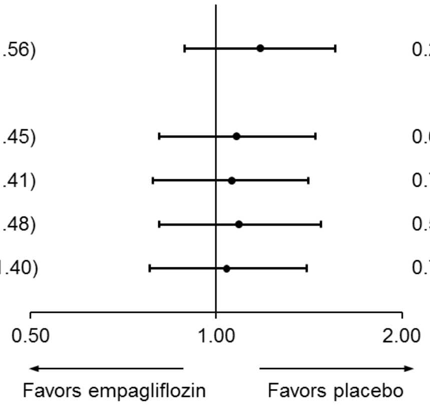

*Events observed from randomization to the end of the study in treated set (patients treated with ≥1 dose of study drug) (pre-specified).

†Events observed during treatment or ≤90 days, ≤30 days or ≤7 days after a patient's last intake of trial medication in treated set (patients treated with ≥1 dose of study drug) (post-hoc).

‡Events that occurred during treatment or ≤30 days after a patient's last intake of trial medication in patients who received study drug for ≥30 days (cumulative) (pre-specified).

HR, hazard ratio. CI, confidence interval

Supplementary Figure II. Time to transient ischemic attack. (A) Pre-specified modified intent-to-treat analyses in the treated set* and (**B**) Post-hoc on-treatment analysis in treated set + 90 days†. Cumulative incidence function. Hazard ratios are based on Cox regression.

A Modified intent-to-treat analyses in the treated set*

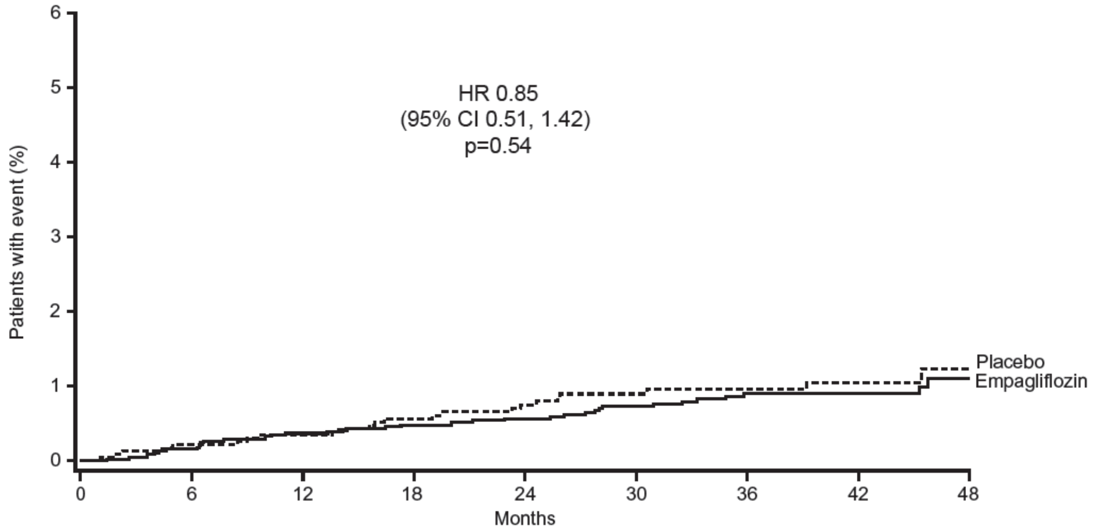

No. of patients	0	6	12	18	24	30	36	42	48
Empagliflozin	4687	4620	4537	4456	4024	2976	2519	1658	401
Placebo	2333	2288	2248	2192	1958	1451	1231	795	172

B Sensitivity analysis in treated set + 90 days†

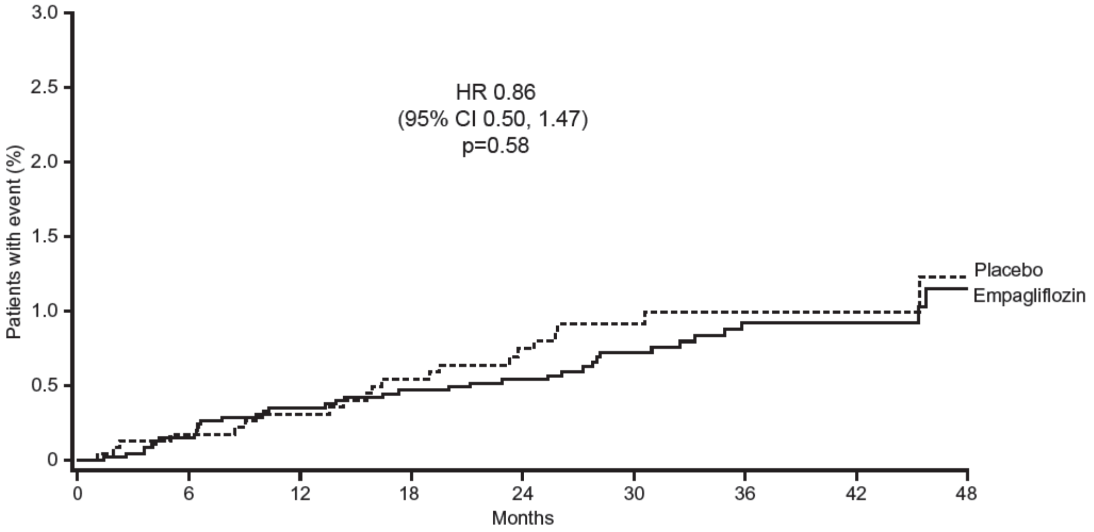

No. of patients	0	6	12	18	24	30	36	42	48
Empagliflozin	4687	4474	4277	4104	3612	2615	2183	1435	343
Placebo	2333	2235	2108	1994	1706	1225	1006	631	130

*Events observed from randomization to the end of the study in treated set (patients treated with ≥1 dose of study drug). †Events observed during treatment or ≤90 days after a patient's last intake of trial medication in treated set.

HR, hazard ratio. CI, confidence interval.

Supplementary Figure III. Hazard ratios for time to first stroke with empagliflozin compared with placebo by baseline HbA1c in deciles. Post-hoc Cox regression analyses.

	Patients with event/analysed		HR	(95% CI)
	**Empagliflozin**	**Placebo**		
All patients	164/4687	69/2333	1.18	(0.89, 1.56)
Baseline HbA1c				
≤7.1%	16/598	10/283	0.73	(0.33, 1.61)
>7.1 to ≤7.3%	10/437	10/208	0.48	(0.20, 1.14)
>7.3 to ≤7.5%	15/428	6/237	1.33	(0.52, 3.43)
>7.5 to ≤7.7%	22/441	10/205	0.98	(0.46, 2.06)
>7.7 to ≤8.0%	17/624	6/308	1.41	(0.56, 3.59)
>8.0 to ≤8.2%	10/369	3/192	1.76	(0.48, 6.38)
>8.2 to ≤8.5%	16/473	10/253	0.83	(0.38, 1.83)
>8.5 to ≤8.8%	17/382	3/206	3.19	(0.94, 10.90)
>8.8 to ≤9.2%	20/473	8/210	1.17	(0.52, 2.66)
>9.2 to ≤12.1%	21/460	3/231	3.43	(1.02, 11.50)

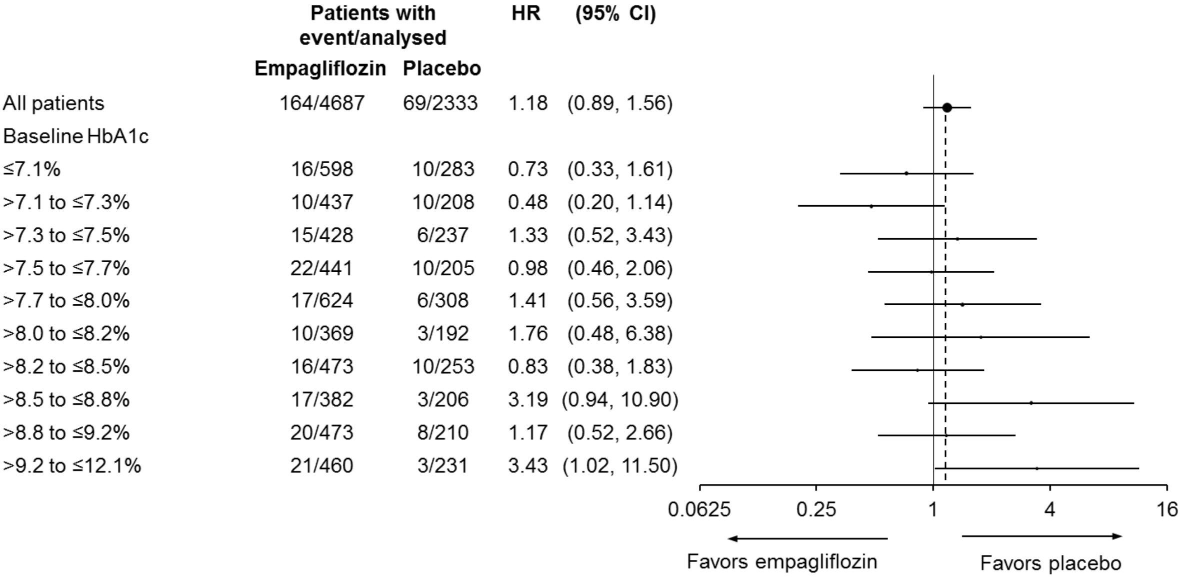

Treated set (patients treated with ≥1 dose of study drug). Two patients were excluded from this analysis as baseline HbA1c was not available.
p=0.1920 for treatment by baseline HbA1c percentile category interaction
HR, hazard ratio. CI, confidence interval.

Definitions of Transient Ischemic Attack and Stroke

Transient ischemic attack (TIA)

TIA: a transient episode of neurological dysfunction caused by focal brain, spinal cord, or retinal ischemia, without acute infarction.

Stroke

Stroke: the rapid onset of a new persistent neurologic deficit attributed to an obstruction in cerebral blood flow and/or cerebral hemorrhage with no apparent non-vascular cause (e.g., trauma, tumor, or infection). Available neuroimaging studies are considered to support the clinical impression and to determine if there is a demonstrable lesion compatible with an acute stroke. Strokes are classified as ischemic, hemorrhagic, or unknown.

Diagnosis of stroke.

For the diagnosis of stroke, the following 4 criteria should be fulfilled:

- Rapid onset of a focal/global neurological deficit with at least one of the following:
  - Change in level of consciousness
  - Hemiplegia
  - Hemiparesis
  - Numbness or sensory loss affecting one side of the body
  - Dysphasia/aphasia
  - Hemianopia (loss of half of the field of vision of one or both eyes)
  - Other new neurological sign(s)/symptom(s) consistent with stroke

NOTE: If the mode of onset is uncertain, a diagnosis of stroke may be made provided that there is no plausible non-stroke cause for the clinical presentation

- Duration of a focal/global neurological deficit ≥24 hours OR < 24 hours if this is because of at least one of the following therapeutic interventions:
  - Pharmacologic (i.e., thrombolytic drug administration)
  - Non-pharmacologic (i.e., neurointerventional procedure [e.g. intracranial angioplasty])

OR

  - Available brain imaging clearly documents a new hemorrhage or infarct

OR

  - The neurological deficit results in death

- No other readily identifiable non-stroke cause for the clinical presentation (e.g., brain tumor, trauma, infection, hypoglycemia, peripheral lesion)
- Confirmation of the diagnosis by at least one of the following:*

  - Neurology or neurosurgical specialist
  - Brain imaging procedure (at least one of the following):
    - CT scan
    - MRI scan
    - Cerebral vessel angiography
  - Lumbar puncture (i.e. spinal fluid analysis diagnostic of intracranial hemorrhage)

If a stroke is reported but evidence of confirmation of the diagnosis by the methods outlined above is absent, the event will be discussed at a full CEC meeting. In such cases, the event may be adjudicated as a stroke on the basis of the clinical presentation alone, but full CEC consensus is mandatory.

If the acute focal signs represent a worsening of a previous deficit, these signs must have either

- Persisted for more than one week

OR

- Persisted for more than 24 hours and were accompanied by an appropriate new CT or MRI finding

Classification of stroke

Strokes are sub-classified as follows:

- Ischemic (non-hemorrhagic): A stroke caused by an arterial obstruction due to a thrombotic (e.g., large vessel disease/atherosclerotic or small vessel disease/lacunar) or embolic etiology. This category includes ischemic strokes with hemorrhagic transformation (i.e. no evidence of hemorrhage on an initial imaging study but appearance on a subsequent scan)
- Hemorrhagic: A stroke due to a hemorrhage in the brain as documented by neuroimaging or autopsy. This category includes strokes due to primary intracerebral hemorrhage (intraparenchymal or intraventricular) and primary subarachnoid hemorrhage
- Not assessable: The stroke type could not be determined by imaging or other means (e.g., lumbar puncture, neurosurgery, or autopsy) or no imaging was performed

Clinical Events Committee Charter for Assessment of Ischemic Stroke

Classification of the subtype of ischemic stroke:

- Large-artery atherosclerosis
  - Studies/findings should show significant stenosis or occlusion of a major brain artery or branch cortical artery, [presumably due to atherosclerosis]
  - Studies should exclude potential sources of cardiogenic embolism

- Brain imaging findings/diagnostic studies
  - Computerised tomography (CT) or magnetic resonance imaging (MRI)
    - Cortical or cerebellar lesions and brain stem or subcortical hemispheric infarcts greater than 1.5 cm in diameter
  - Duplex imaging or arteriography
    - Stenosis of greater than 50% of an intracranial or extracranial artery
    - If these studies are normal or show only minimal changes, this diagnosis cannot be made.
- Clinical findings
  - Cerebral cortical impairment (including, but not limited to aphasia, neglect, restricted motor involvement)

    OR
  - Brain stem or cerebellar dysfunction
  - History of intermittent claudication, transient ischemic attacks, carotid bruit or diminished pulses

- Cardioembolism
  - Studies should exclude potential large artery atherosclerosis sources of thromboembolism or embolism
  - History of conditions listed on the high risk and medium risk categories
  - Brain imaging/clinical findings
    - Refer to large artery atherosclerosis clinical and brain image findings
    - Must identify at least 1 cardiac source for an embolism.
    - Must have evidence of a prior transient ischemic attack (TIA) or stroke in more than one vascular territory or system embolism supports a clinical diagnosis of stroke.
  - A medium-risk cardiac source with no other cause of stroke is a ‘possible cardioembolic stroke’
  - Cardioembolism sub-categories
    - High-risk cardioembolism
    - Mechanical prosthetic valve
    - Mitral stenosis with atrial fibrillation

        - Atrial fibrillation (other than lone atrial fibrillation)
        - Left atrial/atrial appendage thrombus
        - Sick sinus syndrome
        - Recent myocardial infarction (<4 weeks)
        - Left ventricular thrombus
        - Dilated cardiomyopathy
        - Akinetic left ventricular segment
        - Atrial myxoma
        - Infective endocarditis
    - Medium-risk cardioembolism
        - Mitral valve prolapse
        - Mitral annulus calcification
        - Mitral stenosis without atrial fibrillation
        - Left atrial turbulence (smoke)
        - Atrial septal aneurysm
        - Patent foramen ovale
        - Atrial flutter
        - Lone atrial fibrillation
        - Bioprosthetic cardiac valve
        - Nonbacterial thrombotic endocarditis
        - Congestive heart failure
        - Hypokinetic left ventricular segment
        - Myocardial infarction (>4 weeks, <6 months)

- Small-vessel occlusion (lacune)
    - There should be no evidence of cerebral cortical dysfunction, no potential cardiac sources for embolism.
    - Large extracranial arteries should not have a >50% stenosis of an ipsilateral artery.
    - Brain imaging findings
        - CT or MRI

        - Should be normal
          OR
        - Brain stem or subcortical hemispheric lesion with a less than 1.5 cm diameter.

  - Clinical findings
    - 1 traditional clinical lacunar syndrome
    - history of diabetes mellitus or hypertension.

- Stroke of other determined etiology
  - Studies should exclude large artery atherosclerosis and cardiac sources of embolism
  - Brain Image findings and clinical findings
    - Demonstrate clinical symptoms of stroke
    - CT or MRI findings of an acute stroke (regardless of size/location)
    - Blood tests or arteriography should support a rare cause of stroke such as nonatherosclerotic vasculopathies, hypercoagulable statue or hematologic disorders.

- Stroke of undetermined etiology
  - Two or more causes identified (therefore unable to make final determination)
  - Negative evaluation (available information present and complete)
  - Incomplete evaluation (missing information/incomplete information)

A response to probable or possible will be chosen for all categories except undetermined

- Probable: A "probable" diagnosis is made if the clinical findings, neuroimaging data, and results of diagnostic studies are consistent with one subtype and other etiologies have been excluded.
- Possible: A "possible" diagnosis is made when the clinical findings and neuroimaging data suggest a specific subtype but other studies are not done.

References in Supplementary Appendix

1. Adams HP, Jr., Bendixen BH, Kappelle LJ, Biller J, Love BB, Gordon DL, et al. Classification of subtype of acute ischemic stroke. Definitions for use in a multicenter clinical trial. TOAST. Trial of Org 10172 in Acute Stroke Treatment. Stroke 1993;24:35-41.
