# Supplementary material for: Empagliflozin and Cerebrovascular Events in Patients With Type 2 Diabetes Mellitus at High Cardiovascular Risk
Source: Stroke. 2017 Apr 24;48(5):1218–25. doi: 10.1161/STROKEAHA.116.015756 (PMC5404404; doi:10.1161/STROKEAHA.116.015756)
Supplement: Supplementary file 2 [file str-48-1218-s002.pdf]

Advertisement
Opsumit
macitentan
Learn more ▶
Prescribing Information

Acknowledgment Permission Form

Journal: Stroke

Manuscript No.: STROKE/2016/015756

First author: Zinman, B

Title of work: Empagliflozin and Cerebrovascular Events in Patients with Type 2 Diabetes at High Cardio

Acknowledgment Permission:

Authors must provide written permission/approval from all individuals mentioned by name in the Acknowledgements section of a submitted manuscript. By signing this form, any and all acknowledged persons therefore state that they have read and approved the mention of their names in the Acknowledgment section of the aforementioned paper.

Printed Name: Elizabeth Ng

Signature: [illegible]

Date: 06/01/2017

Printed Name: Wendy Morris

Signature: [illegible]

Date: 06/01/2017

Printed Name:

Signature:

Date:

Printed Name:

Signature:

Date:

TREND WATCH NOW AVAILABLE!
Trend Watch

American Heart Association. American Stroke Association.
LOOK who's talking

Advertisement
In pulmonary arterial hypertension (PAH)...
LOOK
OUTCOMES
OUTCOMES
OUTCOMES
long term
Should the decision for long-term PAH treatment be based on short-term trial results?
LEARN MORE
ACTELION
November 2015 15014931
© Actelion Pharmaceuticals, Ltd
